# Prognostication of papillary thyroid microcarcinoma based on preoperative ultrasound

**DOI:** 10.3389/fendo.2023.1101705

**Published:** 2023-01-30

**Authors:** Samuel M. Cohen, Julia E. Noel, Michael Baroody, Lisa A. Orloff

**Affiliations:** Department of Otolaryngology-Head and Neck Surgery, Stanford University School of Medicine, Stanford, CA, United States

**Keywords:** papillary thyroid microcarcinoma, active surveillance, ultrasound, papillary throid carcinoma, thyroid nodular disease

## Abstract

**Background:**

Diagnosis of papillary thyroid microcarcinoma, defined as papillary thyroid carcinoma measuring 1cm or less in greatest diameter, has increased with improvements in ultrasound technology and widespread familiarity and utilization. Given the indolent course of papillary thyroid carcinoma, active surveillance is considered an acceptable alternative to surgical resection for select patients. Candidacy for active surveillance is determined by a number of patient and tumor characteristics. Specifically, the location of the tumor within the thyroid gland plays one of the key roles in decision making. Here we evaluate characteristics of the primary tumor and distance to the thyroid capsule in association with locoregional metastases to help guide risk assessment.

**Methods:**

Retrospective chart review of all thyroid surgeries performed by two surgeons at one medical center from 2014-2021 to evaluate characteristics of papillary thyroid microcarcinoma on preoperative ultrasound that are associated with locoregional metastatic disease.

**Results:**

Our data show a sensitivity of 65% and specificity of 95% for identifying regional metastases in papillary thyroid microcarcinoma using preoperative ultrasound. We found no correlation between regional metastasis and size of tumor, distance to thyroid capsule or trachea, tumor contour, or presence of autoimmune thyroiditis. Nodules in the superior or midpole were associated with central or lateral neck metastases, whereas nodules in the isthmus or inferior pole were only associated with central neck metastases.

**Conclusions:**

Active surveillance may be a reasonable option for even those papillary thyroid microcarcinomas adjacent to the thyroid capsule.

## Introduction

Papillary thyroid microcarcinoma (PTMC) is defined by the World Health Organization as papillary thyroid carcinoma (PTC) with a maximum diameter of 1 cm or less. The prevalence of PTMC has dramatically increased in the past fifty years largely with the evolution of ultrasound technology and ultrasound-guided FNA (1). While rates of PTMC have risen throughout the world, rates of mortality from thyroid cancer have remained stable, raising concern for overtreatment (2–4).

Accordingly, the traditional treatment modality of surgical resection for PTMC has been reconsidered. Prospective clinical trials from Japan demonstrate that active surveillance (AS) is a safe option for management of low-risk (T1aN0M0) PTMC (5, 6). Professional organizations in Japan and the United States adopted active surveillance as an alternative management strategy in 2010 and 2015, respectively (7, 8).

In 2016, Brito et al. published a clinical framework for risk stratification when considering AS for PTMC (9). This approach considers tumor/neck ultrasound characteristics, patient demographic characteristics, and treatment characteristics, including availability of a multidisciplinary team. To date, several features have been identified that may increase the risk of nodal metastases and impact candidacy for AS, including age <45, male sex, tumor size >5mm, multifocal disease, and extrathyroidal extension (10–13). Location of the primary tumor must also be considered, particularly if growth may affect critical structures or increase surgical risk. Prior study has suggested AS may be appropriate for tumors <7mm or even low-risk PTMCs < 7mm abutting the trachea with an acute angle between the tumor and the tracheal surface, or near the anticipated location of the recurrent laryngeal nerve but with a normal rim of thyroid in the direction of the nerve (14). However, data and validation guiding clinical practice based on preoperative assessment remains limited.

Specifically, in this study we analyze preoperative ultrasound images to identify ultrasound characteristics of PTMC nodules associated with increased risk of regional metastases. Several ultrasound characteristics were identified that may help determine appropriate candidacy for AS.

## Materials and methods

### Study design and patients

This study was approved by the Stanford Institutional Review Board.

A retrospective chart review of all thyroid cancer surgeries performed by two surgeons (J.E.N. and L.A.O.) between 1/1/2014 and 3/1/2021 was performed. All operative and pathology reports were reviewed, and cases were included if a total thyroidectomy or thyroid lobectomy was performed with final pathology indicating PTC equal to or less than 1.0 cm in greatest diameter. Patients were excluded if they had a concurrent PTC >1cm or were undergoing surgery for recurrent disease. Patients with multifocal PTMC were not excluded. In total, 185 PTMC nodules from 158 individuals were identified.

Demographic features (age, gender, race/ethnicity) were collected from the medical record (**Table 1**) and clinical features (indication for surgery, laterality of disease, size of nodule by surgical pathology, presence of adverse features on pathology [extrathyroidal extension, angioinvasion, lymphatic invasion, positive margins], presence of regional metastases, concurrent autoimmune thyroiditis) were collected from preoperative notes, operative reports, laboratory values, and pathology reports. Presence of suspicious lymphadenopathy was collected from preoperative ultrasound reports. To simplify our analysis and optimize statistical power, we combined N1a and N1b into one group. 65 out of 185 PTMC nodules were identified incidentally (resection of PTMC was not the indication for surgery).

**Table 1:** Demographic information for data included in our overall analysis (left column) and in our ultrasound analysis (right column).

	Total (%)	Ultrasound Analysis (%)
**Individuals**	158	84
** Nodules**	185	88
**Age: mean (range)**	50.4 (16-88)	49.0 (23-88)
Sex		
*Female*	117 (74%)	63 (75%)
* Male*	41 (26%)	21 (25%)
Race		
* Asian*	42 (27%)	31 (37%)
* Hispanic*	17 (11%)	7 (8.3%)
* White*	83 (53%)	40 (48%)
* Black*	1 (0.63%)	0 (0%)
* Other*	15 (10%)	6 (7.1%)
**Autoimmune Thyroiditis**	61 (39%)	34 (40%)

**Figure 1:**
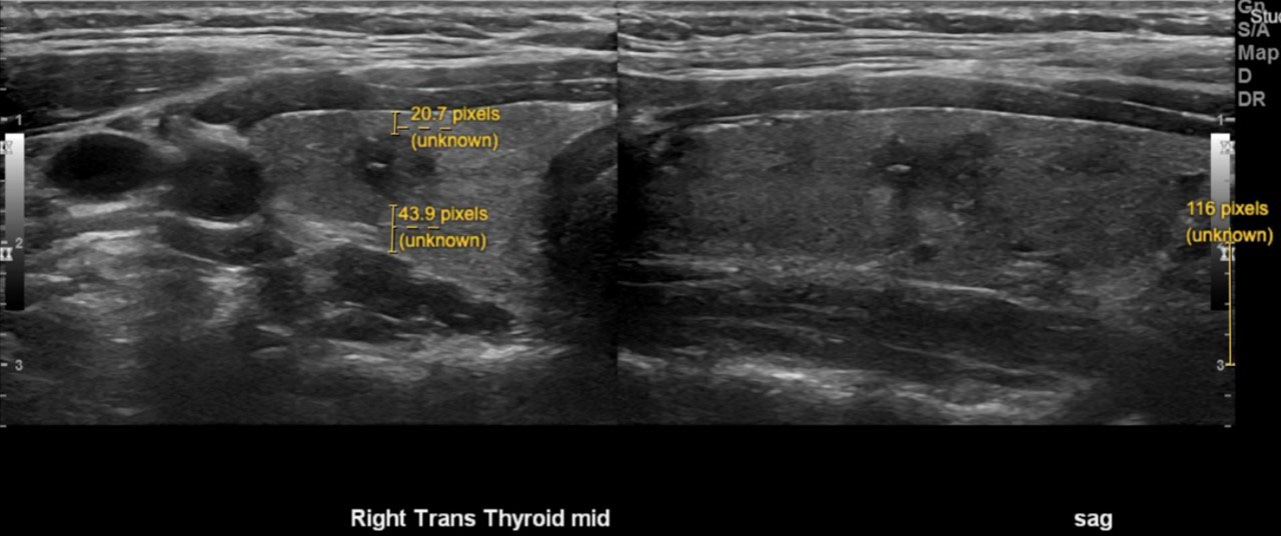
Representative image demonstrating preoperative ultrasound measurements of thyroid nodules. Size of nodule (white). Distance to anterior and posterior capsule (yellow). The pixel size was calibrated for each image using the scale provided by the ultrasound software (bottom right of image).

When available, preoperative ultrasound images were reviewed. Nodules were included in ultrasound analysis if they could be confidently identified on the preoperative ultrasound in one of two scenarios: 1) preoperative FNA confirming PTC, or 2) clearly visible nodule on ultrasound that correlated in size and location with PTMC on surgical pathology without heterogeneous thyroid background or other confounding nodules. 8 out of the 88 nodules used in our ultrasound analysis were identified incidentally. For those nodules included, the following ultrasound features were collected: size, smoothness or lobulation of contour, position within thyroid gland, relationship to trachea, distance to anterior thyroid capsule, distance to posterior thyroid capsule (**Figure 1**). The distance to the anterior and posterior thyroid capsule was independently measured by two of the authors in a blinded fashion (S.M.C. and J.E.N.) and the mean of each of these values was used for analysis.

### Statistical analysis

All statistical analysis was performed using Prism (Graphpad Software, San Diego, CA). Significance tests were two-sided and a p-value <0.05 was considered significant. Analysis of Variance (ANOVA) and t-tests were used to determine if differences between the means of the parameters measured were statistically significant.

## Results

185 PTMC nodules were identified from 158 individuals. 102 nodules (55%) were associated with N0 disease and 46 nodules (25%) were associated with pathologic N1 disease. 37 nodules were associated with Nx disease (no pathological data from lymph nodes available) and were excluded from subsequent analysis. The primary analyses in our study were repeated by combining N0 and Nx data, and no significant differences were found compared to when Nx was excluded (data not shown). For 88 of the original 185 nodules, the PTMC nodule could be confidently identified on preoperative ultrasound images; many nodules were discovered only retrospectively on pathology and we were unable to identify them on preoperative ultrasound due to multinodular disease or small size. 41 of these 88 nodules were associated with N0 disease and 33 nodules were associated with N1 disease. 14 nodules were associated with Nx disease and were excluded from ultrasound analysis.

**Figure 2:**
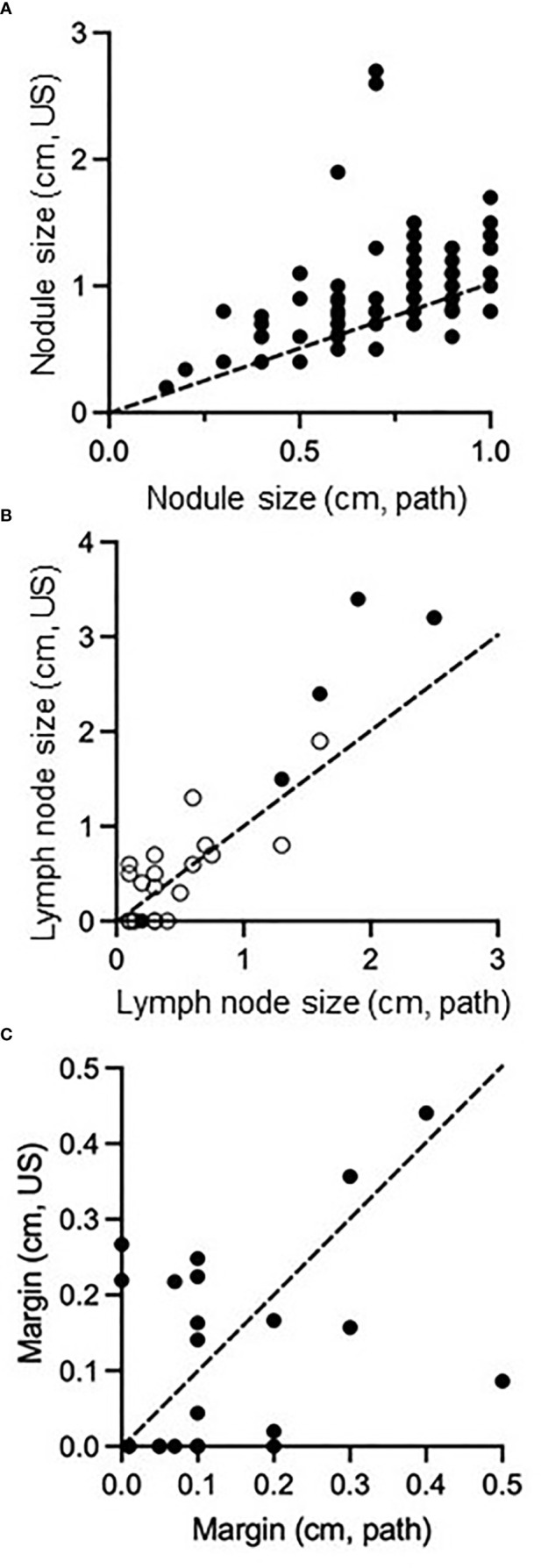
Comparison of size of PTMC nodules **(A)**, size of regional metastatic disease **(B)**, and distance of tumor margin from thyroid capsule/surgical resection boundary **(C)** as measured on preoperative ultrasound compared to surgical pathology. Dashed line passes through (0,0) and (1,1), representing equivalent size on both measurements. In **(B)**, open circles represent central neck nodes and closed circles represent lateral neck nodes.

Appropriate management of thyroid disease requires accurate assessment of nodule size. In our data set, nodules ranged in size from 0.2cm to 2.7cm on preoperative ultrasound and from 0.15cm to 1.0cm on pathology (**Figure 2A**). As PTMC is defined by surgical pathologic size, our dataset contains nodules with ultrasound dimensions greater than 1cm that still ultimately classify as PTMC. 57 nodules (65%) were 1cm or less on preoperative ultrasound and 31 nodules (35%) were >1cm. When ultrasound and pathology values for each nodule (**Figure 2A**) or metastatic lymph node (**Figure 2B**) were compared, most values fell above a line with a slope of 1 passing through (0,0). Ultrasound measurements *in vivo* tended to exceed the size of disease compared with *ex vivo* histopathology. We next analyzed the distance between nodules and the thyroid capsule. There was a poor correlation between distance measured on ultrasound and pathologic margins (**Figure 2C**, R squared = 0.17). There was no significant difference in distance to thyroid capsule (measured on ultrasound) between thyroid nodules with a pathologic margin of <5mm or >5mm (p>0.05). Pathologic margin size was not reported when it exceeded 5mm, limiting sample size for analysis of this characteristic.

**Figure 3:**
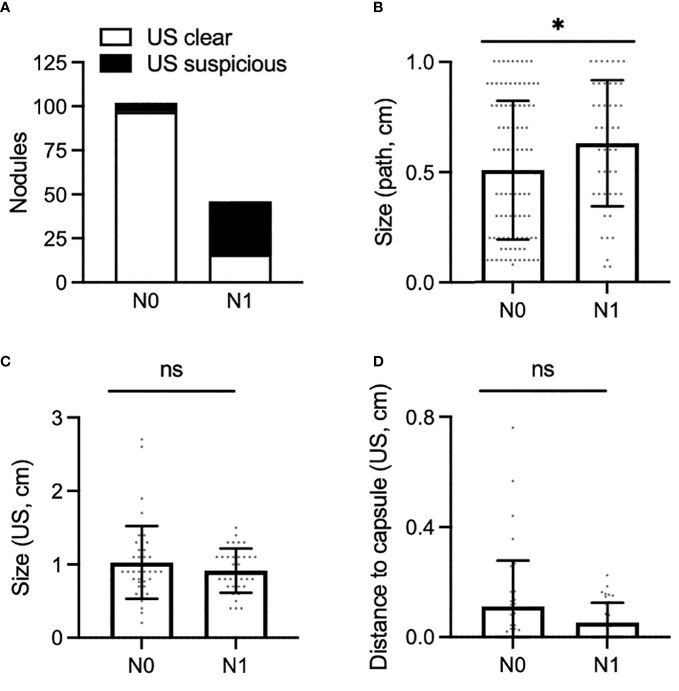
**(A)** Proportion of PTMC nodules associated with N0 neck (left) and N1 neck (right), for which the preoperative ultrasound was clear of disease (white bar) or was suspicious for pathologic nodes (black bar). **(B,C)** Size of PTMC nodules associated with N0 or N1 disease as measured on surgical pathology **(B)** and preoperative ultrasound **(C)**. **(D)**, Shortest distance to the anterior or posterior thyroid capsule as measured on preoperative ultrasound in PTMC nodules associated with N0 or N1 disease. *, p < 0.05. ns, not stastistically significant.

Preoperative ultrasound is a valuable tool to assess for regional metastasis. We analyzed the success rate of ultrasound in accurately capturing nodal disease, compared to final pathology (**Figure 3A**). Among patients with N1 disease, the preoperative ultrasound accurately predicted suspicious lymph nodes in 30 out of 46 of PTMC nodules; we report a sensitivity of 65% for preoperative ultrasound to rule out regional metastasis in PTMC. Among patients with N0 disease, preoperative ultrasound was free of visible lymphadenopathy in the central neck in 97 out of 102 cases, corresponding to a specificity of 95% for confirming regional metastasis.

Understanding which factors may predispose to regional metastasis is an area of significant clinical interest. On examination of pathology reports, PTMC nodules associated with N1 disease were larger than those associated with N0 disease (**Figure 3B**, mean: N0 [0.51cm], N1 [0.63cm], p<0.05). In contrast to histopathological data, however, preoperative ultrasound did not capture any difference in size of nodules associated with N0 vs N1 disease (**Figure 3C**, mean: N0 [1.03cm], N1 [0.92cm], p>0.05). There was also no difference in rate of metastatic disease amongst PTMC nodules measuring ≤5mm vs >5mm. This result was consistent when size was measured on ultrasound (p >0.05) or on final pathology (p>0.05).

We next determined whether distance of a nodule to the thyroid capsule may increase the likelihood of metastasis. Distance of each nodule to the anterior and posterior thyroid capsule was measured, and the smaller of these two measurements was used for analysis; no difference was seen between nodules associated with N0 or N1 disease (**Figures 1** and **3D**, mean: N0 [0.11cm], N1 [0.0.5cm], p>0.05). Of note, 34 of 88 of nodules (39%) were adjacent to either the anterior or posterior thyroid capsule, measured as a minimum distance to the capsule of 0cm. Extrathyroidal extension was identified on pathology for 2 of 88 PTMC nodules (2%), one of which abutted the posterior thyroid capsule. Positive margins were identified on pathology for 2 of 88 PTMC nodules (2%), but were distinct from the 2 nodules with extrathyroidal extension.

Previous data suggest that observation is safe for certain PTMCs abutting the trachea (14).

**Figure 4:**
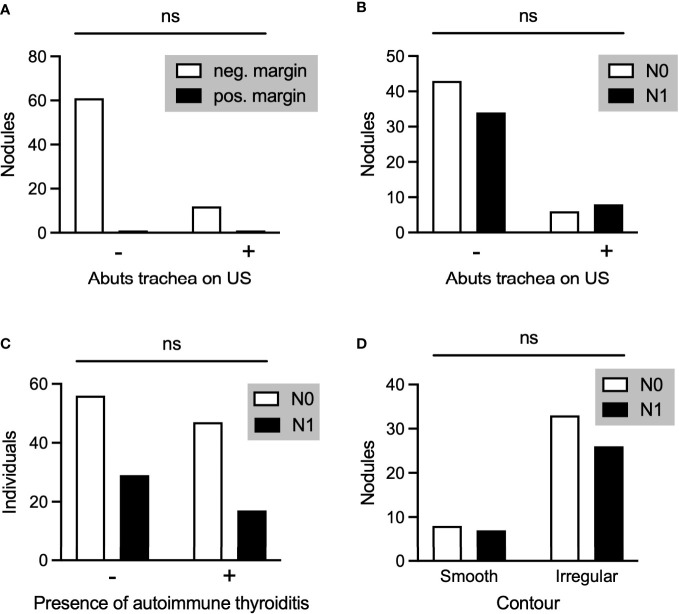
**(A)** PTMC nodules associated with negative or positive pathological margins in those nodules that did (+) or did not **(-)** abut the trachea on preoperative ultrasound. **(B-D)** Comparison of PTMC nodules associated with N0 or N1 disease with regard to whether the nodule abutted the trachea **(B)**, the individual had autoimmune thyroiditis **(C)**, and the contour of the nodule **(D)**. ns, not stastistically significant.

In our data set, of 13 PTMC nodules abutting the trachea, only 1 was associated with positive margins on pathology and none with extrathyroidal extension. There was no difference between the rate of positive margin (**Figure 4A**) or the rate of metastatic disease (**Figure 4B**) between those nodules abutting the trachea and those not abutting the trachea, although analysis was limited by small sample size. Ito and colleagues have described certain “high risk” characteristics of PTMC nodules abutting the trachea, including an obtuse angle with relation to the trachea and a size larger than 7mm (14). Consistent with those data, our single nodule with positive margins had an obtuse angle and was larger than 7mm. Of the remaining 12 nodules, 10 had acute angles, 1 had an obtuse angle, and 1 had a 90 degree angle with regard to the trachea. 6 of these nodules were 7mm or larger and 6 nodules were smaller than 7mm.

**Figure 5:**
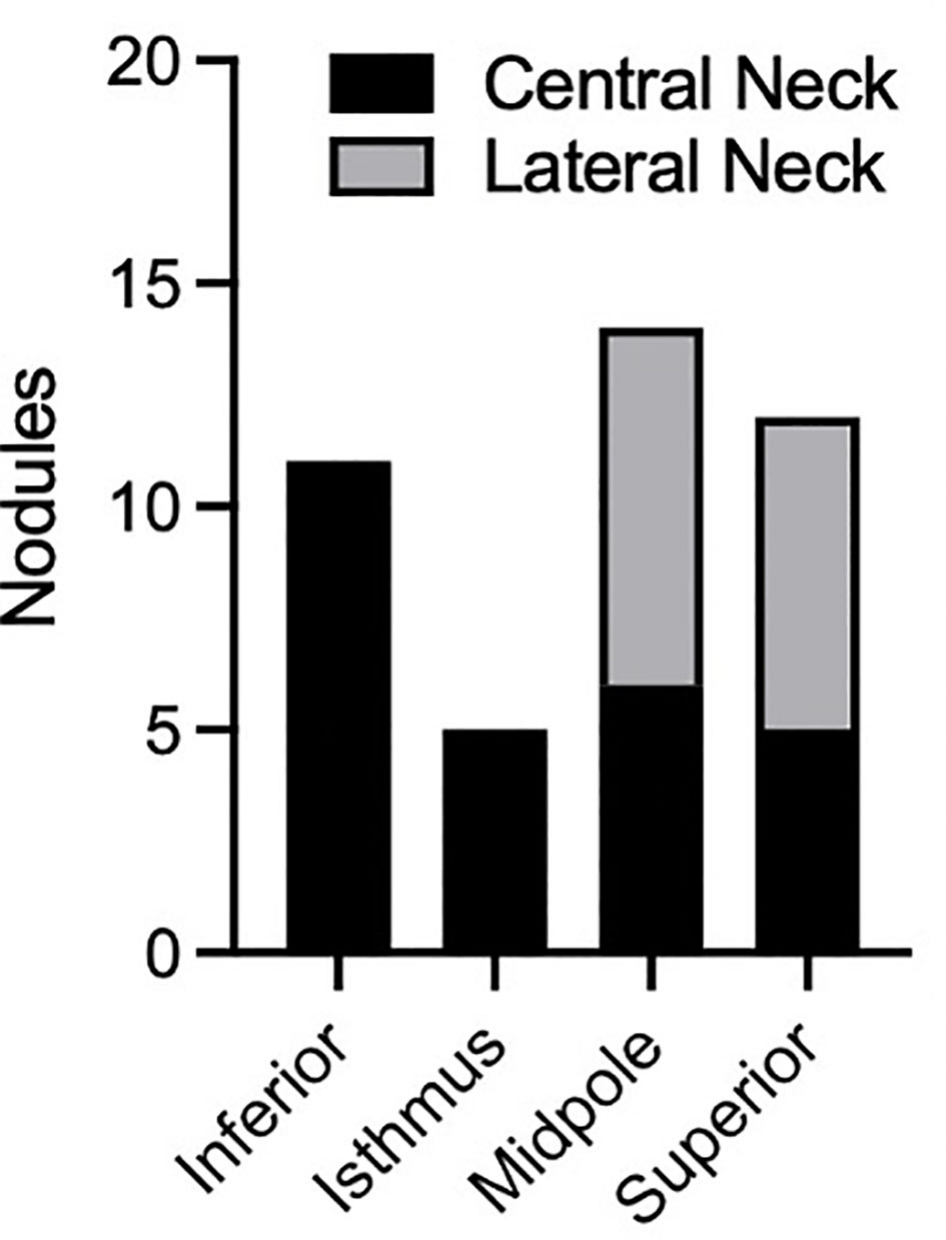
Analysis of PTMC nodules with regional metastasis. Plot displays location of nodules within the thyroid and location of notal metastasis (central or lateral neck). In TNM staging, central and lateral neck metastases are referred to as N1a and N1b disease, respectively.

Finally, we analyzed several additional factors which have been suggested to be associated with risk of metastases from PTMC. Presence or absence of autoimmune thyroiditis was not associated with a difference in rates of metastatic disease amongst patients with PTMC (**Figure 4C**). Furthermore, there was no difference in rates of metastatic disease associated with PTMC nodules with irregular as opposed to smooth contours (**Figure 4D**). The location of PTMC nodules within the thyroid, however, did show differing results. While nodules found in the superior or mid thyroid pole were significantly associated with both central and lateral neck metastases, those in the isthmus and inferior pole were associated only with central metastases (**Figure 5**).

## Discussion

The question of which PTMCs are candidates for observation is increasingly pressing as acceptance of active surveillance has become mainstream. Our data demonstrate that ultrasound measurements overestimate nodule size compared to final pathology, which is the metric by which PTMC is defined. As one third of PTMC nodules in our data set measured greater than 1cm on preoperative ultrasound, an ultrasound size cutoff of 1.5cm or 2cm rather than a pathology size cutoff of 1cm may be more clinically relevant for future studies. Nonetheless, our study provides informative data to help guide the decision-making process in determining surveillance candidacy.

We found a sensitivity of 65% and specificity of 95% for identifying PTMC regional metastases, consistent with prior data. Two recent meta-analyses reported ultrasound sensitivity for identifying central and lateral neck metastases of 28-33% and 70-73%, respectively, with a specificity of 93-95% and 84-89%, respectively (15, 16). The discrepancy in sensitivity between our study and prior data likely relate to our combining central and lateral neck metastasis.

Considering the relatively poor sensitivity to rule out regional, especially central, metastases, identification of ultrasound characteristics of high risk PTMC nodules is paramount. Our data suggest that proximity to or contact with the thyroid capsule alone should not necessarily discourage a physician from offering active surveillance (**Figure 3D**). This finding is consistent with one retrospective study of 1,622 patients (12); however, a meta-analysis of 43,750 patients found that >25% PTMC contact with the thyroid capsule was significantly associated with lateral neck metastases (13). Within our dataset, nodule contour was classified as smooth or irregular based on ultrasound, but there was no association between PTMC contour and regional metastases. Previous meta-analysis found that PTMC contour and also shape (taller than wide) conveyed no increased risk of lateral neck metastases (13). Together, these data suggest that PTMC nodules regardless of contour may be safely observed. This analysis also suggests that some PTMC nodules abutting the trachea may be safely observed, as described in Ito et al. (14) This prospective study of 1143 patients with PTMC concluded that nodules with an obtuse angle to the trachea should undergo surgical resection. However, many nodules may be observed, including those <7mm even if abutting trachea, those ≥ 7 mm with an acute angle to the trachea, and those with a normal rim of thyroid tissue separating the nodule from the course of the recurrent laryngeal nerve (14).

Prior data suggest that PTMC >5mm are higher risk for regional lymph node metastases than those ≤5mm (11, 12). We caution against relying on size of PTMC as measured on ultrasound. While there may be differences in pathological size, such slight variations in size may not be reliably captured on clinic ultrasound. Our cohort contained few nodules ≤5mm, which is not surprising considering the difficulty of accurately assessing and performing biopsy on such small nodules, and the discouragement of intervening on such small lesions. Furthermore, we found no relationship between mean size on ultrasound and regional metastasis. Thus, none of our results refute prior data or recommendations that nodules ≤5mm in size may safely be observed. One caveat is the assumption that such nodules, even if malignant, are PTMC. It is important to remember that medullary thyroid carcinoma is in the differential diagnosis and has overlapping sonographic features with PTC. While rare, medullary carcinoma should be considered during the history-taking, physical examination, and possible laboratory testing of patients with thyroid nodules.

Our data did demonstrate the tendency of superior pole nodules to metastasize to the lateral neck, and inferior thyroid nodules to metastasize to the central neck. This finding is consistent with a prior meta-analysis (13) and with the anatomy of the lymphovascular drainage pathway of the thyroid gland. Considering that N1b disease has a higher mortality than N1a for patients over 55 yo, surgery may be considered more strongly for superior/midpole PTMCs in this age group, compared to isthmus or inferior pole nodules. Regardless, ultrasonography of the entire neck, including bilateral central and lateral compartments, is warranted during the ultrasound evaluation and surveillance of PTMC.

There was no correlation between the presence of autoimmune thyroiditis and identification of metastatic disease. In general, data on the relationship between autoimmune thyroid disease and the extent of disease is variable. For example, a meta-analysis of over 15,000 patients reported that, although the presence of thyroiditis increased the risk of harboring PTC, it protected against lateral neck metastasis (17), whereas another large meta-analysis of 43,750 patients found no association with metastatic disease (13). Given the conflicting data, the presence of thyroiditis is not an independent factor in determining candidacy for active surveillance.

## Limitations

This study was limited by a small sample size; we designed the study primarily to include preoperative ultrasound analysis of nodules as this was lacking in the literature. We chose to exclude Nx disease to increase clarity of results, but by doing so our sample size was further decreased. Other limitations are the retrospective nature of the study, the lack of preoperative cytologic confirmation of 65/185 PTMC nodules included in our study, and the lack of follow-up data for recurrence or survival.

## Conclusions

For patients with PTMC, the sensitivity of preoperative ultrasound to rule out regional metastasis in PTMC, albeit potentially micro metastasis, is mediocre. Surgery is recommended when ultrasound identifies frank extrathyroidal extension or lymph node metastases, or when adverse clinical features are present such as vocal fold paralysis or active increase in nodule size on interval exam. Younger age (less than 45 years) is also associated with long-term disease progression and favors intervention (10). Many other tumor and demographic factors, however, have less clear implications on candidacy for surveillance, and there remains insufficient data for strong recommendations. The data presented here suggest that active surveillance may be a reasonable option for even those papillary thyroid microcarcinomas adjacent to the thyroid capsule, certain nodules abutting the trachea, and those with irregular margins, regardless of autoimmune disease status, in the absence of the risk factors noted above. Nevertheless, and as previously reported, candidates for observation should be managed by an experienced multidisciplinary team with the capability of high-quality ultrasonography and a mechanism to ensure proper follow up.

## Data availability statement

The raw data supporting the conclusions of this article will be made available by the authors, without undue reservation.

## Ethics statement

The studies involving human participants were reviewed and approved by Stanford Institutional Review Board. Written informed consent for participation was not required for this study in accordance with the national legislation and the institutional requirements.

## Author contributions

SC: Devised study outline, collected patient data, analyzed data, wrote manuscript. JN: Devised study outline, collected patient data, analyzed data, wrote manuscript. MB: Wrote manuscript. LO: Devised study outline, collected patient data, wrote manuscript. All authors contributed to the article and approved the submitted version.
